# Pain dilates time perception

**DOI:** 10.1038/s41598-017-15982-6

**Published:** 2017-11-16

**Authors:** Amandine E. Rey, George A. Michael, Corina Dondas, Marvin Thar, Luis Garcia-Larrea, Stéphanie Mazza

**Affiliations:** 1 EMC Laboratory, University Lyon2, Lyon, France; 2 Petre Andrei University, Iași, Romania; 3 Central Integration of Pain, Lyon Neuroscience Research Center, Inserm U1028 & UCB Lyon 1, Lyon, France

## Abstract

We have all experienced that time seems stretched during unpleasant situations. While there is evidence of subjective time overestimation when perceiving external unpleasant stimuli, no study has measured the dilation of time when individuals experience an unpleasant situation in their own body. Here we measured the time dilation induced by a painful homeostatic deviance using temporal bisection task. We show that being in pain leads to an expansion of subjective time whereby a stronger increase in pain perception relative to non-painful stimulation leads to a stronger time-estimate distortion. Neurophysiological studies suggest that time estimation and the perception of self might share a common neural substrate. We propose that, along with bodily arousal and attentional capture, the enhancement of self-awareness may be critical to support dilated subjective time when experiencing pain. As other homeostatic deviances, pain may induce a focus on ourselves contributing to the impression that “time stands still”.

## Introduction

The subjective passage of time is not isomorphic to physical time – time flies during pleasant experiences but drags on during unpleasant situations^1^. The subjective evaluation of time is influenced by a number of factors including emotion^2–4^, attention^5^ and anxiety^6^. While pain is known to stretch subjective time, its effect on the perceived duration of external events has not been investigated so far^7^. A growing body of evidence suggests that the experience of pain is also linked to time. For example, increasing the duration of a nociceptive stimulus also increases pain - a well-known mechanism called ‘temporal summation’^8,9^. Reciprocally, the subjective intensity of experimental pain can be reduced when its perceived duration is artificially shortened, while the actual duration is kept constant^10^. The control of time, for example by explicitly stating how much time is remaining, is a good strategy to help children cope with acute procedural pain and discomfort^11^. However, very few studies so far have assessed the opposite, i.e. whether the duration of pain can, by itself, generate subjective time distortions, or time expansion. Studies showed that pain influences the retrospective estimation of duration in such a way that subjects judge a previous painful situation as being longer than it actually was, in both clinical^4,12^ and experimental conditions^13^. However, retrospective evaluation imply that participants are not told beforehand that they will have to estimate time duration, and consequently their *a posteriori* assessment is based on memory processes. Hence, the influence of pain on time estimation should be rather explored in prospective paradigms involving an “experienced duration” tested in real-time^14^. Indeed, prospective time estimation is both closer to the actual state of the individual, and less related to memory and coping processes than retrospective estimations. Here we used a psychophysical paradigm to measure in real-time the distortion of time estimation induced by tonic pain during a temporal bisection task. Participants were instructed to determine whether sequential visual stimuli was rather short or rather long as compared to a “template” short and long duration previously learned, while immersing their hand in water at neutral or painful temperatures. Pain significantly lengthened the subjective duration of stimuli presented concomitantly and the more heightened was the pain perceived, the more time was overestimated. We therefore propose that pain, like other unpleasant homeostatic deviations (e.g., hunger or thirst) leads to the impression that “time stands still”.

## Material and Methods

### Participants

Forty undergraduates (28 females) with normal or corrected-to-normal vision volunteered to take part in the experiment (*M*_age_ = 25.04, *SD* = 3.62). Exclusion criteria were the existence of a chronic or pre-existing pain condition and taking painkillers. The study protocol was approved by the local ethics Committee (Lyon Sud Est 3, 2015-010B, EUDRACT 2014-A01280-47) and carried out in accordance with the relevant guidelines and regulations. Written informed assent and consent were obtained from all the participants. A power analysis based on a pilot study confirmed that, given a desired power of 0.80 and an alpha level of 0.05, a minimum sample size of 33 participants was sufficient to detect a difference.

### Material and stimulation procedure

Presentation, timing and control of visual targets were performed on a 21.5-inch Apple IMac using OpenSesame 2.9.7^15^. Each participant was tested individually during a session that lasted approximately 50 minutes. The stimulus was a grey square (RGB code: #646464) viewed under an angle of 9.1° and displayed in the center of the computer screen with variable durations. For the cold pressor test, two containers were used (50 × 39.5 × 32.9 cm), both containing water, one at 12 °C (53,6 °F–pain condition) and the other at room temperature of about 25 °C (77 °F–control condition). The water was continuously in movement to avoid warming around the immersed hand, and its temperature was controlled throughout the test.

### Temporal bisection task

During a *training phase*, participants were introduced to the short and long presentation durations of the grey square. Participants fixed their gaze on a fixation point for 500 ms and then observed the grey square appearing in the center of the screen, which could last for a short (250 ms) or long (750 ms) presentation period. Participants learned to categorize the duration as “short” or “long” by pressing the corresponding key (down arrow key for the “short” responses and up arrow key for the “long”). There were 12 trials for each duration with an inter stimulus interval (ISI) of one second. To ensure that the participants had clearly understood the task, they received feedback for the first 4 trials.

**Figure 1:**
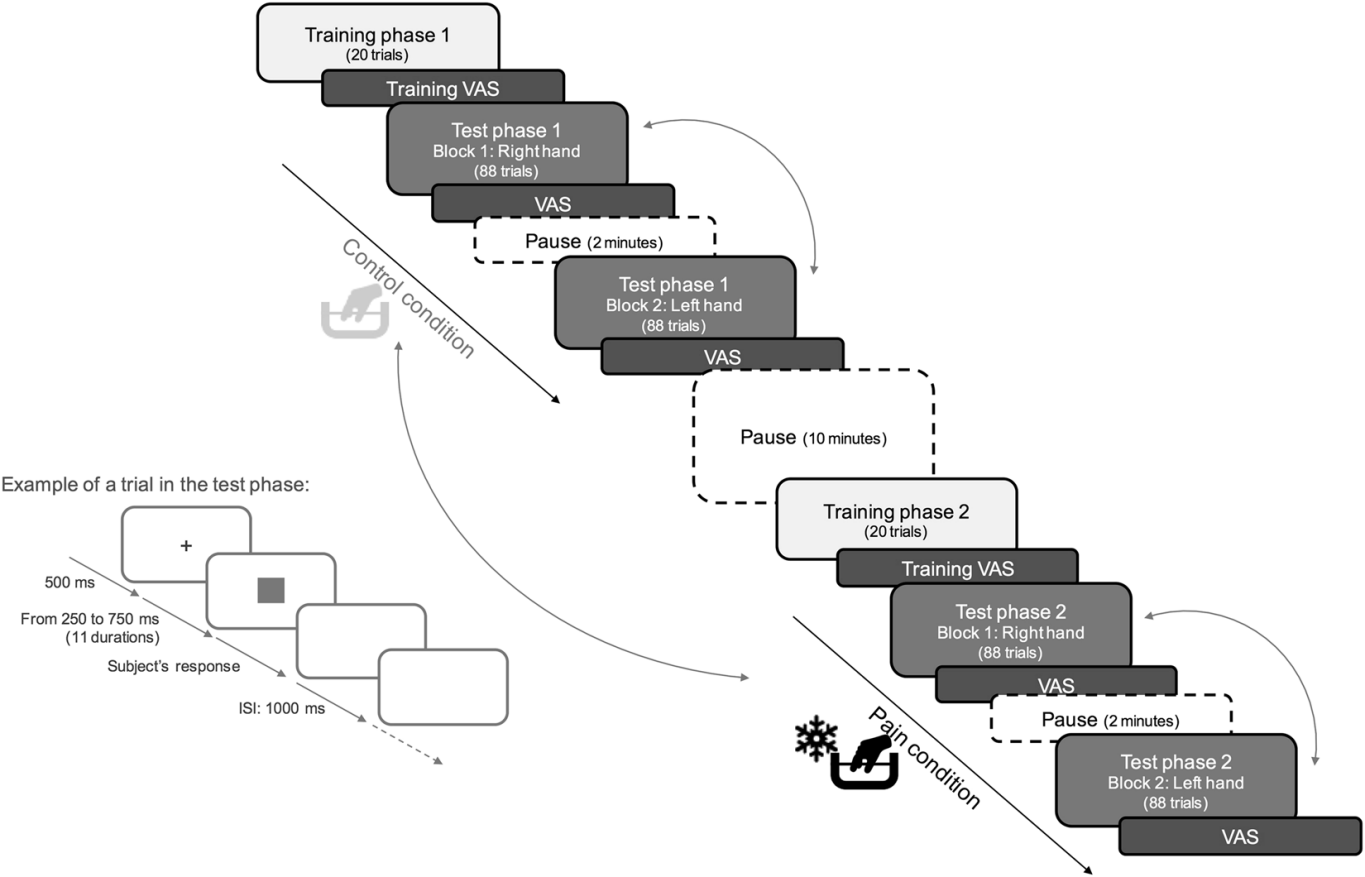
Illustration of the procedure with the two training phases and the two test phases. In the training phases, participants learned to discriminate the ‘short’ (250 ms) and ‘long’ (750 ms) durations. In the test phases, participants were instructed to categorize a series of grey squares as ‘rather short’ or ‘rather long’ based on the two templates previously learned. The test phases were divided in two blocks: one block with the right hand in the water and one block with the left hand in the water. A trial in the test phase corresponded to: a fixation point for 500 ms, a grey square presented during 250, 300, 350, 400, 450, 500, 550, 600, 650, 700 or 750 ms and a blank screen until subject’s response. The stimulus duration was random and equiprobable. The Inter Stimulus Interval was 1000 ms. The pain and control conditions were counterbalanced as well as the hand immersed in the water (representing by the curved arrows). Pain intensity was assessed using a Visual Analogue Scale (VAS) at the end of each block.

During the *test phase*, subjects had to categorize a series of stimuli as ‘rather short’ or ‘rather long’ based on the two templates used during the training phase. Thus, the grey square was presented during 250, 300, 350, 400, 450, 500, 550, 600, 650, 700 or 750 ms in random order, with ISI of one second and a fixation point between two consecutive stimuli. The participants immersed their hand in tepid water (control condition) or cold water (pain condition) while performing the time estimation task of visual targets. A two-condition (pain vs. control) within-subject design was used. The order of conditions was randomized, half of the participants starting with the control condition. Each condition was divided in two blocks: half of the participants begun with their right hand and continued with their left hand, and vice versa for the other half. Each target duration was presented eight times per block. To avoid the hand becoming numb, each condition was split into two blocks of 3–4 minutes each, using alternating hands. The first hand immersed in cold water was therefore retrieved at the end of the block, the subsequent block being performed with the other hand after a 2-minute break. Participants could remove their hand from the water but they were encouraged to place it back again as soon as possible. The pain condition and the control condition were separated by a 10-minute break (Fig. 1). A total of 352 trials were presented (11 durations × 8 presentations × 2 blocks × 2 conditions).

To ensure that the unpleasantness resulting from the water at room temperature in the control condition did not induce a time distortion, we ran a complementary experiment in which the participants had to put their hand in an empty container (see supplementary data).

### Pain evaluation

Pain intensity was assessed using a visual analog scale (VAS) presented on the screen, formed by a 622 pixels horizontal line with six schematic faces^16^ from no pain in the left (“aucune douleur” in French) to excruciating pain in the right (“douleur intolérable” in French). Participants indicated their response by clicking on the line. The analog scores were converted to a 0- to 10-point scale. VAS were presented at the end of the training phases and at the end of each block of the test phases (two scores for the pain condition and two scores for the control condition). A pain score for each condition (pain and control) was calculated by averaging the scores obtained at the end of each block.

### Statistical analysis

Initial data processing and subsequent analyses were performed using RStudio version 3.2.2 (R Foundation for Statistical Computing). Raw responses were converted into proportions of ‘long duration’ responses per participant and condition (i.e. the proportion of responses where the participant classified the target duration as being ‘long’, irrespective of its actual duration). The data were plotted against the actual duration of the stimulus, and fitted locally using the “*model-free”* statistical package^17^. This representation allows illustrating a systematic bias toward longer estimations by a leftward shift of the function (the subjects will more often classify the duration of the visual stimulus as ‘long’). The shifting of the function (i.e. the stimulation duration giving rise to 50% of “long” responses and 50% of “short” responses) was calculated for each subject. The difference between the bisection point in pain and control conditions were compared using a bilateral paired Student *t*-test (with Cohen’s d for the effect size). This analysis was also conducted on the just noticeable difference (JND) as a measure of sensitivity of the temporal bisection task. The pain scores were compared using bilateral independent or paired Student *t*-tests.

For all analyses, a bilateral *p* value of 0.05 was used as the criterion for statistical significance. Means and standard errors are given for each condition.

## Results

Participants were instructed to determine whether a visual stimulus was “rather short” or “rather long” as compared to a template short and long duration previously learned, while immersing their hand in water at neutral (control condition) or painful (pain condition) temperatures. Among the 40 participants, two were removed from the analysis because of function with flat slopes. These two participants were also the two only who removed their hand twice during the cold pressor test.

### Temporal bisection task

**Figure 2:**
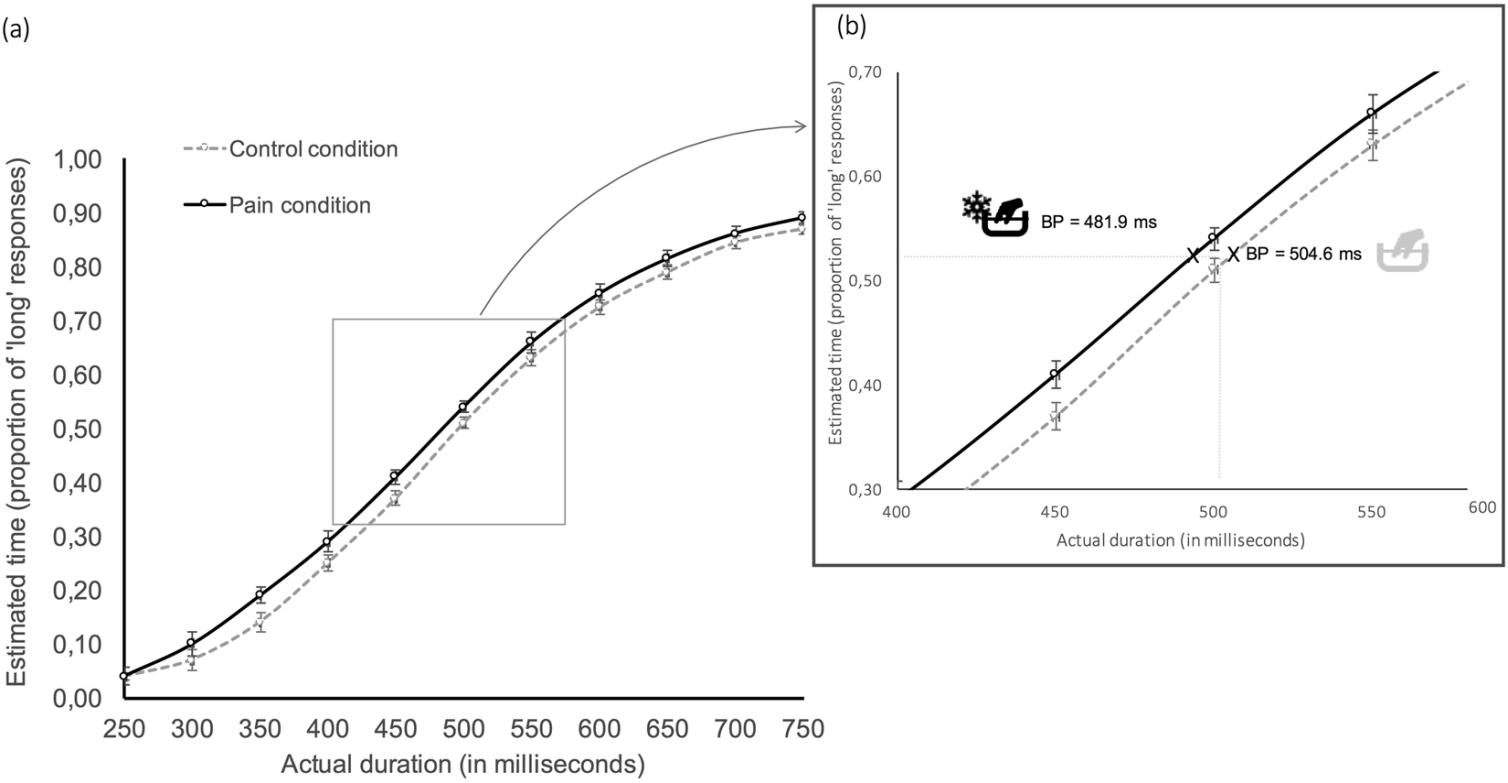
(**a**) Mean proportion of long responses plotted against actual duration from 250 ms to 750 ms between the pain (solid line) and the control (broken line) conditions. (**b**) A focus on the bisection point (BP) in each condition, i.e. the duration giving rise to 50% of “long” and “short” responses. The bisection point shifted towards the left in the pain condition, consistent with a lengthening effect in the pain condition compared to the control condition. Errors bars represent standard errors corrected for within-subject design.

The proportion of responses estimated to be of ‘long duration’ was significantly higher in the pain condition compared to the control condition (*t*(37) = 3.26, *p* = 0.003, *d* = 0.32). The whole sigmoid bisection function in the pain condition was shifted towards the left, indicating that subjects overestimated time when they were in pain across all durations tested (Fig. 2).

There was a significant difference between the bisection point (i.e. the duration giving rise to 50% of “long” and “short” responses) between the pain and the control conditions, *t*(37) = 3.19, *p* = 0.0029, *d* = 0.34. Indeed, the bisection point shifted from 504.6 ± 10.6 ms in the control condition, to 481.9 ± 10.8 ms in the pain condition, consistent with a lengthening effect when the participants were in pain (see supplementary data). On average, the time needed for a stimulus to be considered ‘long’ was 20 ms less during pain than in the absence of pain. Complementary analyses did not reveal any significant effect of the stimulated side (left vs right, *p* = 0.37) or of the order of conditions (first pain vs first control, *p* = 0.85). Comparison of the sensitivity index (JND) between pain (91.8 ± 4.9) and control (95.2 ± 5.8) conditions revealed no statistical difference (*t*(37) = 0.84, p = 0.41).

### Pain evaluation and the way pain changes the perception of time

To ensure that no pain sensation induced by the cold pressor test remained after the 10-min pause, we compared VAS scores obtained at the end of the second training phase. Results showed no significant difference between the VAS scores collected after the control condition (0.74 ± 0.10) and after the pain condition (0.82 ± 0.18), *t*(29.45) = 0.41, *p* = 0.68, indicating that there was no carry-over effect from the pain condition.

During the test phases, participants gave higher scores in the painful (5.35 ± 0.34) than control condition (1.98 ± 0.27) (t(37) = 12.86, p < 0.001, d = 1.77). In order to show that a shift towards higher scores of pain co-occurred with a shift towards smaller bisection points (i.e., a lengthening of perceived time) in the temporal bisection task, an alpha regression coefficient was computed (α = covariance(x,y)/variance(x) where x is the pain scores collected with the VAS and y the bisection points in the temporal bisection task) expressing decrements of bisection points per change in the VAS score. The mean alpha was −4.53 ± 1.69; it was reliably different from zero (t(37) = 2.68, p < 0.01, d = 0.43). In other words, whenever participants felt more pain, they perceived a lengthening of time.

## Discussion

Pain significantly lengthened the subjective duration of visual stimuli presented concomitantly. Moreover, stronger increases in pain perception relative to non-painful stimulation led to stronger time-estimate distortions. Conversely, during the non-painful control condition, participants estimated time very accurately (deviating only by 0.5–1.6% from its actual duration), thus eliminating a general, non-specific bias.

Over the last decades, the most conspicuous models of time perception have hypothesized the existence of a pacemaker–accumulator clock representing internal time^18,19^. The pacemaker of this assumed clock produces a series of “time units”, or “pulses”, the accretion of which determines the experienced duration. Thus, a higher number of pulses emitted per unit of time may lead to a subjective impression of time lengthening. Within this framework, our results reflect an excessive accumulation of time units when participants are in pain, relative to the control condition. The reason why ‘time units’ may accumulate differentially remains very imperfectly known. According to the Scalar Expectancy Theory [SET,^20^], arousal has been hypothesized to be a crucial factor responsible for the speeding up of the internal clock, and hence of time dilation, and indeed a number of studies have shown that time is subjectively stretched by a variety of arousal modifiers such as body temperature^21^, pharmacological substances^22,23^ or emotions^3,24,25^. Pain, as any unpleasant, emotionally negative and arousing stimuli, may lead to a time overestimation by increasing the pacemaker rate. It is classically assumed that arousal is a component of the pain response [for a review,^25^] and arousal can modulate pain threshold^26^. However, differential effects on pain perception can emerge under identical arousal levels, as a function of the positive or negative valence of associated stimuli^27^, and several studies using emotional stimuli showed that arousal was not the only candidate to explain time distortion in these contexts^2,28^. Indeed, for a same level of arousal, positive and negative emotional stimuli have shown opposite effects on subjective time; consequently an interaction between valence and arousal, rather than a direct effect of the latter, has been postulated as crucial for time estimation^2^. In our experiment, the nociceptive signal processing, the negative valence of pain, and the arousing effect induced by the painful stimulation probably interacted to contribute to time distortion.

Pain not only enhances arousal, but also impacts the direction of attentional processes^29,30^. Subjective time tends to accelerate when attention is directed to attention-capturing external events or internal cognitions, whereas it drags on when cognitive appeal is lacking^14^ (e.g., boredom), when attention orients to the time estimation itself^7^ or when homeostatic deviations prompt attention to reorient towards one’s own body, like with pain or hunger. The stretching of subjective time experienced during pain in this experiment may be understandable within such framework: repeated pain-induced attentional shifts toward the painful body would be at the basis of time distortion through enhancing the accumulation of ‘time units’. In support of this view, in our study, higher scores of pain co-occurred with a lengthening of time perception.

Directing attention toward the experience of time may also imply orienting attention towards the self. A close connection between time perception and interoceptive self-oriented processes has been shown experimentally^31–33^, leading Wittman^34^ to quote Martin Heidegger’s proposal “I measure myself as I measure time”^35^. Being in pain reflects a deviation from homeostasis which threatens integrity and prompts repeated conscious access to one’s self. Such consciousness of one’s suffering body relates to the concept of “sentience” (in the sense of the ability of being self-aware), which, along with bodily arousal and attentional capture, are thought to build prospective time estimation^2,14,24,36^.

Studies in patients have not identified single focal lesions specifically affecting the perception of time^37^, the neural bases of which appear to depend on distributed cortico-subcortical networks^38^. This network largely overlaps with that involved in self-awareness [review in^34^]. In particular, behavioral and imaging studies suggest that the anterior insular cortex, which integrates bodily pain signals, is a critical core-component involved in pain integration, self-awareness and the sense of time^39–41^. The exact nature of the pacemaker, or pacemakers, putatively producing impulses when detecting the above cortical activations remains however an open question. The most prominent neurobiological model of time estimation, the striatal beat-frequency model, considers that iterative cortical activation patterns can be detected by basal ganglia spiny neurons, whose repetitive firing rate would allow estimating subjective time^42,43^.

## Conclusion

As documented and quantified in the present study, being in pain leads to the impression that “time stands still”. Pain, as all unpleasant situations, increases arousal and has attention grabbing properties. In the specific case of pain, homeostatic deviance also increases self-awareness by directing attention to the body. The commonalities between brain activities underlying time estimation and self-consciousness suggest that reorienting attention to the body may contribute to the dilation of pain experience. Such enhanced ‘sentience’, together with bodily arousal and attention capture, might build up prospective time estimation. Future studies are needed to disentangle the weight of each of these mechanisms in subjective time perception.

## Electronic supplementary material

Supplementary data
