# Supplementary material for: Pain dilates time perception
Source: Sci Rep. 2017 Nov 16;7:15682. doi: 10.1038/s41598-017-15982-6 (PMC5691055; doi:10.1038/s41598-017-15982-6)
Supplement: Supplementary file 1 — Supplementary data [file 41598_2017_15982_MOESM1_ESM.pdf]

Pain dilates time perception

Amandine E. Rey, George A. Michael, Corina Dondas, Marvin Thar, Luis Garcia-Larrea, and Stéphanie Mazza

Supplementary data

To ensure that the unpleasantness resulting from the water at room temperature in the control condition did not induce a time distortion, we run a complementary experiment in which the participants had to perform the temporal bisection task with their hand in an empty container.

Method

Twenty-four participants (18 females, M_{age} = 26.82, *SD* = 4.03), different from the main study, performed the temporal bisection task with their hand in an empty container. Exclusion criteria were the existence of a chronic or pre-existing pain condition and taking painkillers. All participants stated that they did not experience pain at the beginning to this complementary experiment.

The procedure was the same as the main study, the only difference was the absence of a cold pressor test in the test phase: to mimic the position of the arm and the hand of the pain and control conditions, participants placed their hand in an empty container with the arm along their body. Participants took part to one test phase for a total of 176 trials.

Results

Temporal bisection task

The independent Student *t*-test analyses conducted on the bisection points revealed no significant difference between the results of this complementary experiment (503.6 ± 6.8) and those of the previous control condition with water at room temperature (504.6 ± 10.6), $t(57.98) = -0.08$, $p = .53$. The somatic sensation induced by the water at room temperature was not unpleasant enough to lengthen

subjective time. As expected, a significant difference between the results of this complementary experiment and those of the previous pain condition (481.8 ± 10.8) was observed, $t(57.71) = 1.71$, $p = .047$, $d = .41$.

Pain evaluation

Even with the hand in an empty container, participants showed a pain score higher than 0 (1.45 ± 0.20), and there was no statistical difference between this complementary experiment and the previous control condition (hand in ambient water, 1.98 ± 0.27), $t(59.782) = 1.45$, $p = .15$. As expected, there was a significant difference between the VAS scores of the complementary experiment and those of the previous pain condition (hand in cold water, 5.35 ± 0.34), $t(60.229) = 9.19$, $p < .001$, $d = 2.22$).
